# Neural processing of prototypicality and simplicity of product design in forming design preferences

**DOI:** 10.1371/journal.pone.0297148

**Published:** 2024-01-19

**Authors:** Erin Cho, Shin-Ae Yoon, Hae-Jeong Park

**Affiliations:** 1 School of Fashion and Textiles, The Hong Kong Polytechnic University, Kowloon, Hong Kong; 2 Department of Media and Communication, Konkuk University, Seoul, South Korea; 3 Department of Cognitive Science, Yonsei University, Seoul, South Korea; 4 Department of Nuclear Medicine, Department of Psychiatry, Graduate School of Medical Science, Brain Korea 21 Project, Yonsei University College of Medicine, Seoul, South Korea; 5 Institute of Human Complexity and Systems Science, Center for Systems and Translational Brain Sciences, InYonsei University, Seoul, Republic of Korea; University of Technology Sydney, AUSTRALIA

## Abstract

The current study investigates the neural correlates when processing prototypicality and simplicity—affecting the preference of product design. Despite its significance, not much is known about how our brain processes these visual qualities of design when forming design preferences. We posit that, although fluency is the perceptual judgment accounting for the positive effects of both prototypicality and simplicity on design preference, the neural substrates for the fluency judgment associated with prototypicality would differ from those associated with simplicity. To investigate these issues, we conducted an fMRI study of preference decisions for actual product designs with different levels of prototypicality and simplicity. The results show a significant functional gradient between the preference processing of simplicity and prototypicality–i.e., involvement of the early ventral stream of visual information processing for simplicity evaluation but recruitment of the late ventral stream and parietal-frontal brain regions for prototypicality evaluation. The interaction between the simplicity and prototypicality evaluations was found in the extrastriate cortex in the right hemisphere. The segregated brain involvements suggest that the fluency judgment for prototypicality and simplicity contribute to preference choice in different levels of cognitive hierarchy in the perceptual mechanism of the design preference.

## Introduction

Design influences people’s product choices in significant ways. Previous studies have indicated that a positive evaluation of design not only boosts the desire to own a product [1] but also increases the willingness to pay more for it [2]. Furthermore, an appealing design fosters a sense of pride and care in the use of the product, while also aiding in the formation of an individual identity [3]. This phenomenon has expanded the range of products that are appreciated by individual consumers, showcasing the evolving impact of design [4]. The role of product design in consumer choices is becoming more important as functional and technological attributes of alternatives are becoming increasingly homogeneous [5]. Design can affect the overall experience of using a product even when consumed mainly for its functional utilities [1,6].

While the extant literature has demonstrated the critical role of product design in people’s consumption behavior through behavioral experiments and surveys, how our brain processes visual qualities of design when forming design preferences is not fully understood. Past research into the neural mechanisms associated with aesthetic evaluations has primarily focused on how we form preferences for faces [7–10]. Aligning with research on facial preferences, Samizadeh [11] highlights how cultural backgrounds, particularly in East Asian and Western contexts, influence facial morphology perceptions observed in pre-esthetic surgery, shedding light on the varied influences on aesthetic preferences and design evaluations. The scope of research extends beyond faces, encompassing geometric shapes and patterns as well as visual arts like paintings and photographs [12–14], alongside investigations into design creativity design creativity [15,16] and the distinct neural pathways of aesthetic expertise [13]. Additionally, the effects of manipulated digital art production, especially through color saturation, have been a recent focus [17]. Despite its significance, not much attention has been paid to understanding how our brain processes the visual qualities of product design. Our study is conducted to address this void. Although multiple variables could affect one’s preference for design, we focus on identifying the neural bases for the two critical visual properties of product design: prototypicality and simplicity.

Prototypicality, also known as typicality, refers to how well an object exemplifies a category, or its alignment with the average or most common attributes of that category [18,19]. There is a widespread tendency to favor prototypical stimuli over more atypical ones, a preference known as the ’beauty-in-averageness’ effect. A classic example of this effect is the preference for faces that are considered average or prototypical [18,20]. The underlying reason for this effect is argued to be driven by our innate inclination to view prototypicality as an indicator of potential mating value [21]. This perception is linked to attributes such as sexual characteristics, health status, and personality traits [22]. Research spanning various natural and man-made categories, ranging from animals to consumer products like watches and cars, has demonstrated the favorable impact of prototypicality on aesthetic preferences, similar to its effect on perceptions of human faces [23–27]. These studies suggest the existence of broader cognitive processes that influence the preference for prototypicality beyond just facial perception. The concept of ’fluency’ has been put forward as a key factor in this context [28–30].

Processing ’fluency’ is defined as the ease with which information can be processed [31]. This concept plays a crucial role in human judgment, stemming from the basic principle that processing any form of stimulus demands cognitive effort. The required cognitive work to process a stimulus manifests in the processing speed, accuracy, and the perceived ease or difficulty encountered; this is known as conceptual fluency, but in this context, we refer to it as ’prototypicality’ [32], although Palmer and and colleagues [33] have made a distinction between prototypicality and fluency as separate theories in aesthetic preference. Prototypical stimuli elicit faster responses compared to non-prototypical ones. An example of this is seen when individuals are presented with random dot patterns; prototypical patterns are categorized more rapidly and require less neural effort for perception compared to distorted patterns [34]. This is aligned with the observation of quicker facial electromyography responses when viewing abstract prototypes [35]. Furthermore, the development of prototypicality is understood as a product of category learning in the brain, involving interactions between the basal ganglia and the medial temporal lobe [36]. The effortless and accurate recognition of a stimulus often leads to positive reactions and a favorable evaluation of it [32,34]. That is, people tend to recognize a product design with prototypical visual information with ease, speed, and accuracy, thus liking it more than one with non-prototypical visual information.

Along with prototypicality, visual simplicity, which is defined as the number of elements, also known as perceptual fluency, is another factor that has been shown to affect design preference [37–39] and long been recognized as the significant attribute leading to the positive aesthetic evaluation and judgment of a product [40]. While people’s judgment of preference for simplicity in visual design can depend on several different aspects, such as symmetry, space, clutter, and regularity of elements, the degree of simplicity/complexity is generally represented and operationalized by the amount and intricacy of elements in a visual image [41].

This visual simplicity has also been used to explain our preference for design in the view of fluency. That is, the simpler image is processed more easily, faster, and with greater accuracy, thus generating more pleasure [42–45]. In recent years, the effect of visual simplicity on design preference has been of particular interest in the context of human-computer interactions examining how people evaluate and interact with digital interfaces. For example, researchers [46–49] have highlighted the importance of visual simplicity in aesthetic judgments of website design, especially upon first viewing. This principle is evident with visualization preferences on social media, although exemplified through high-resolution and professional images [50]. Empirical studies have supported that less complex interfaces are preferred because users can comprehend the functions and usability of less complex interfaces with greater fluency [51,52]. People tend to show an aesthetic preference for low redundancy and balanced patterns [53,54] although high redundancy in interface design is often recommended for individuals over the age of 65 [55].

We posit, however, that fluency is the conceptual and perceptual judgment accounting for the positive effects of prototypicality and simplicity on design preference, so the neural substrates for the fluency judgment associated with prototypicality would differ from those associated with simplicity as the mechanisms of fluent processing for prototypicality and simplicity would be different. Studies have indicated that the assessment of prototypicality is based on the degree to which one has been exposed to similar visual information [56] in a given perceptual and conceptual category [19].

This would imply that prototypicality judgment is likely to involve the areas associated with semantic information and top-down processing. On the other hand, the fluency perception related to simplicity/complexity is more likely to start with the amount and intricacy of information that our optical sensory receptors process, which in turn is transferred to the area primarily responsible for processing the first-hand visual information. This means that fluency associated with simple/complex visual information will be governed by the bottom-up process. It is thus likely that processing prototypical/non-prototypical stimuli would activate the multi-regional circuitry of the visual-parietal-frontal and memory-related cortices involved in top-down processing of visual and semantic information [57–59]. Meanwhile, the evaluation of the complexity may primarily be restricted to the primary and secondary visual cortices according to a previous study that showed the involvement of the primary visual cortex and extrastriate cortex (e.g., V3, V4, V5) in the judgment of simplicity/complexity [60,61].

We note that these two aspects should be considered in the context of one’s preferred choice for design. When forming design preference, the cognitive components associated with perceiving prototypicality and simplicity would be involved implicitly, which is the question of interest in the current study of design preference. Specifically, we investigate the cognitive hierarchy in the fluency for simplicity and prototypicality under the preference choice. In so doing, we conducted an event-related fMRI study of preference decisions for actual product designs with scores for prototypicality and simplicity by the participants. To account for the differences caused by product variability, we chose two product categories, chairs and portable speakers for a computer. Unlike some product categories whose shapes, forms, and design elements are relatively standardized, such as TVs, cars, and pans, etc., both chairs and portable computer speakers have a wide range of design variations with differing degrees of prototypicality and simplicity, which character is critical for our study. Using these realistic design materials, we explored differential neural substrates for the fluency judgment associated with prototypicality and simplicity.

## Methods

### Participants

Twenty-two healthy participants were recruited for this study. All participants in the study were selected based on their lack of neurological illness history and were identified as right-handed using a Korean version of Edinburgh Handedness Inventory [62]. The data from three participants were excluded due to low task accuracy (< 50%) across all conditions or missing behavioral data. Thus, the data analysis was conducted with 19 participants (10 males and 9 females). The age range of the participants was between 19 and 30 years old (mean age = 25.18, SD = 3.69). The procedures were conducted in compliance with the guidelines and received approval from the Severance Hospital Institutional Review Board (IRB) at Yonsei University, Seoul, Korea. Data were collected in January 2014 and processed through December 2014; after December 2014, no information that could identify individual participants was accessible to the authors after data collection. The data were collected with written informed consent from participants.

### Stimuli

**Fig 1:**
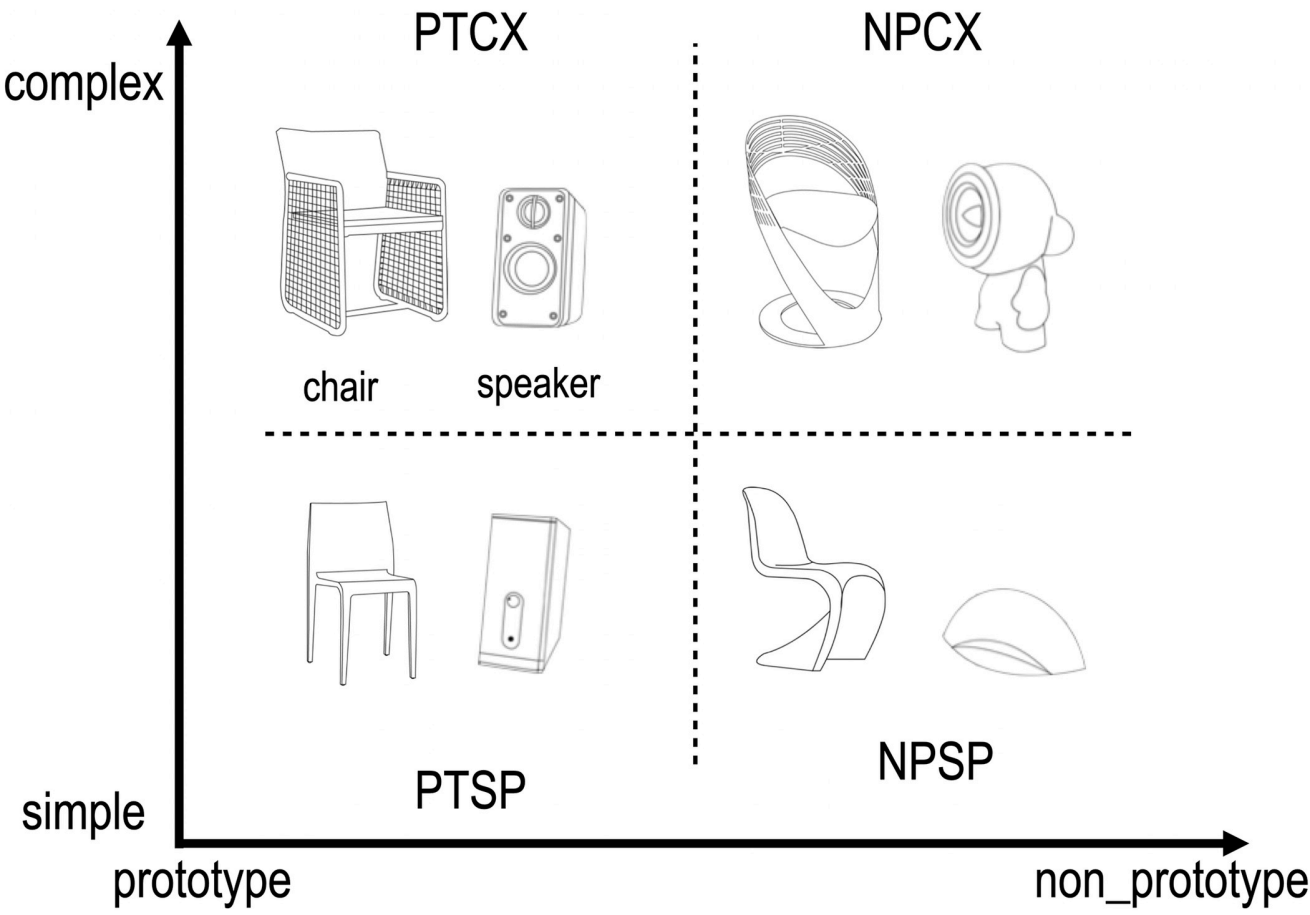
Stimuli consists of two factors considered to influence the product design preference; prototypicality and simplicity. These two factors comprise a total of four conditions described above; NPCX (Non-prototypical Complex), NPSP (Non-prototypical Simple), PTCX (Prototypical Complex), and PTSP (Prototypical Simple). Each stimulus is the representative one that falls in each category (i.e., chair and speaker).

The stimuli of the study were the black-and-white design of chairs and portable speakers for a computer, which were developed as follows. First, we collected about 100 images of chairs and another 100 images of portable computer speakers from websites, magazines, and other print sources. We narrowed the number of images to 136 images (68 images for a chair and 68 images for a speaker) to choose images with differing degrees in terms of prototypicality and simplicity. Next, to control the variances introduced by sizes and colors, (1) all the images were resized to be around 5” x 5” inches in height and width, and (2) only the outline of the shape and design elements of the images were extracted. These redrawn black-and-white images were then presented to the panel of four people with expertise in product design. The expert panel was asked to sort these outline images into four different categories: (1) prototypical and simple (PTSP), (2) prototypical and complex (PTCX), (3) non-prototypical and simple (NPSP), and (4) non-prototypical and complex (NPCX). The intergroup consistency was about 88% across four expert panelists. After removing images whose categorizations were inconsistent and dropping a few more images to have an equal number of stimuli for each category, we finalized the 56 images for chairs and 56 for speakers. More specifically, a set of 14 images was allocated as stimuli for PTSP, PTCX NPSP, and NPCX for both chairs and speakers, totaling 112 stimuli. The representative images of stimuli are presented in **Fig 1**.

### Task procedure

**Fig 2:**
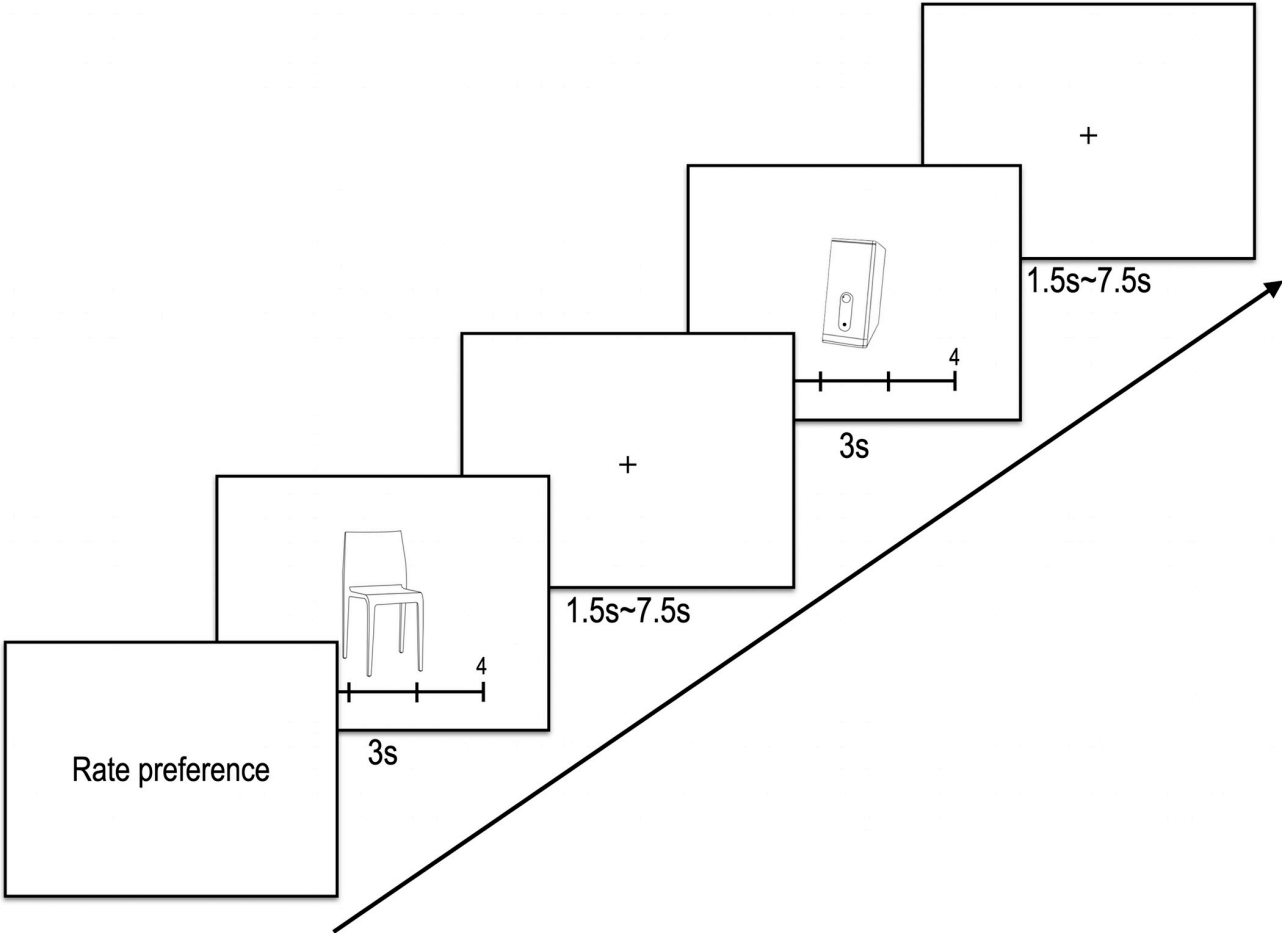
Experiment paradigm. A trial constitutes two stimuli: A product with a preference rating scale below (3 sec) and a cross-fixation point (1.5–7.5 sec). Each experimental condition has a total of 14 trials, so a total of 112 trials (14 trials x 4 conditions x 2 categories) were presented in random order per category in a session. That is, chair stimuli were presented, and then computer speakers were presented randomly.

During the fMRI task, participants were presented with the 112 image stimuli developed for the study in an order optimized for an event-related design using optseq (http://surfer.nmr.mgh.harvard.edu/optseq). The stimuli display was managed using E-Prime software (Psychology Software Tools, USA). Each stimulus was presented for 3000 ms, followed by a cross-fixation point lasting for 1000 to 7500 ms as a jitter (see **Fig 2**). The average interval between trials for the task was approximately 4250 milliseconds. Throughout the fMRI scanning process, participants rated their preference for each image on a 1 (least preferred) to 4 (most preferred) scale, utilizing a response box in their right hand.

### Data acquisition, processing, and statistical analysis

A Philips 3T MRI system (Achieva; Philips Medical System, Best, The Netherlands) was used to scan fMRI axially with a single-shot echo planar imaging (EPI) protocol. The acquisition parameters for fMRI were 2,000 ms repetition time (TR), 30 ms echo time (TE), 90° flip angle, 220 × 220 mm field of view, 128 × 128 recon matrix, 34 in an interleaved sequence, 3.5 mm slice thickness, 0.5 mm slice gap, and thus a 1.719 × 1.719 × 4.0 mm voxel unit. Four dummy scans of fMRI were excluded. Foam padding within the head coil was used to minimize head movement. We acquired a T1-weighted MRI for each subject for spatial processing using a 3D T1-TFE sequence, the acquisition parameters of which are 4.6 ms TE, 9.6 ms, TR, 8° flip angle, 220 mm field of view, a 224 × 224 reconstruction matrix, 0 mm slice gap, and 0.98 × 0.98 × 1.2 mm voxel unit.

Data preprocessing was carried out using Statistical Parametric Mapping (SPM12, http://fil.ion.ucl.ac.uk/spm) [63]. This process involved several steps: firstly, slice timing was applied to the interleaved sequence of images. Next, motion correction was performed by aligning all images to the first image of the sequence. The images were then normalized to the standard Montreal Neurological Institute (MNI) template available in SPM12. Subsequently, the images underwent smoothing using a 6-mm full-width-at-half-maximum (FWHM) Gaussian filter. Additionally, low-frequency drifts in the data were removed by applying a high-pass filter with a cut-off frequency set at 128 seconds.

In the individual-level analysis using the generalized linear model (GLM), we included six motion regressors obtained during the realignment process to mitigate any effects caused by head movements. For group-level activation comparison, we employed a random effect model. The neural activation differences attributable to the two factors (prototypicality and simplicity) were assessed using a flexible design in SPM12, facilitating a repeated measures analysis of variance (ANOVA).

In our group-level analysis, we applied a cluster-level significance criterion. This involved setting a voxel-level threshold at p < 0.005 and an extent threshold of k > 233 voxels. This thresholding approach corresponded to a p < 0.05 significance level, adjusted for multiple comparisons at the cluster level (for full-width half-maximum dimensions of 11 x 11 x 9 mm^3^), as determined by 10,000 Monte Carlo simulations using 3dClustSim in AFNI_17.2.17 [64].

## Results

### Behavioral results

**Fig 3:**
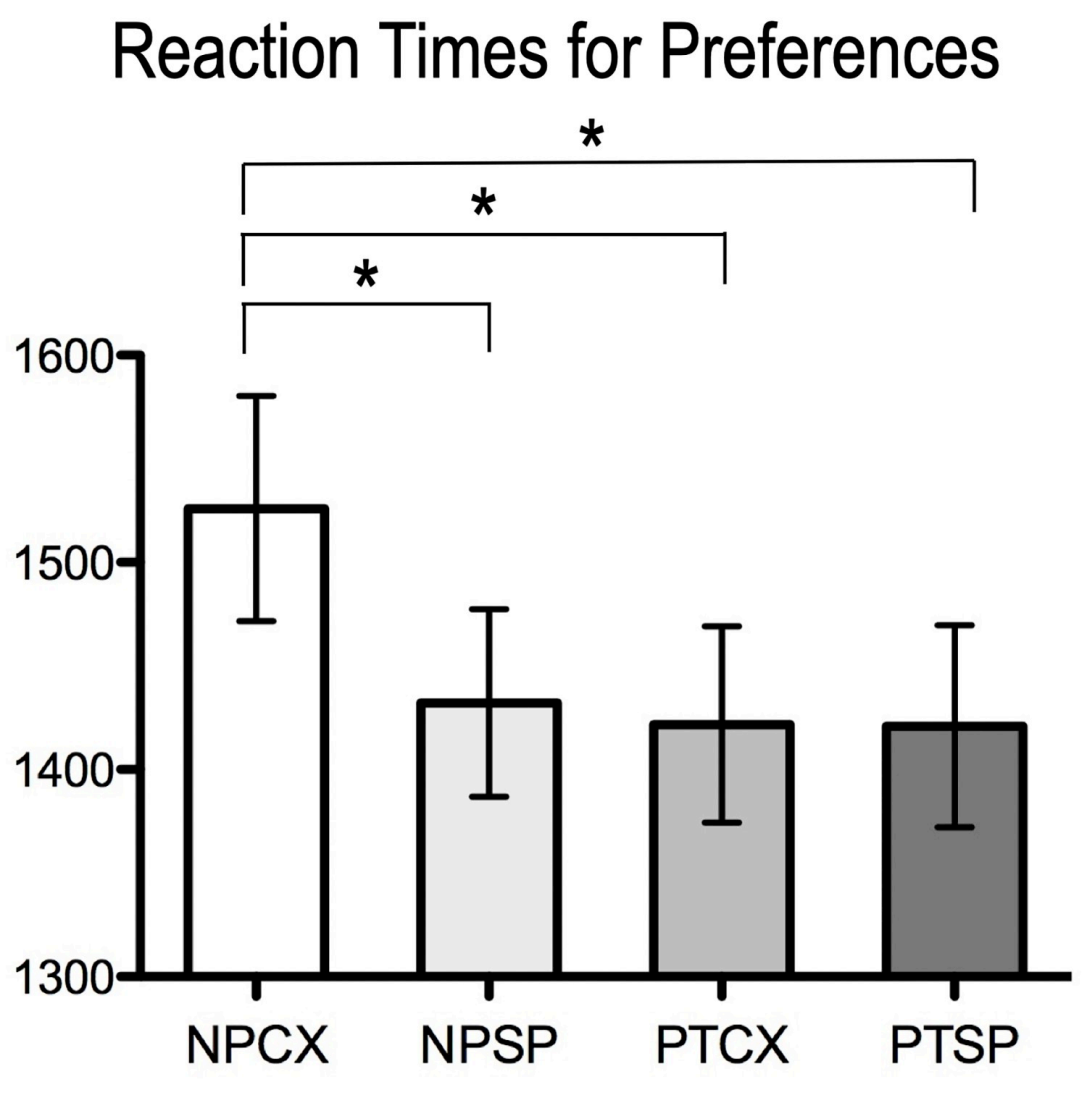
Reaction time for preference rating for all design products; Means and standard errors are displayed.

A 2 x 2 repeated-measures ANOVA with the factors of (non)prototypicality and simplicity (complexity) showed no significant main effects of preference scales and no significant interaction effects between factors. As for the reaction time, however, we found significant main effects caused by prototypicality (*F*_1,37_ = 5.437, *p* = 0.025) and simplicity (*F*_1,37_ = 7.488, *p* = 0.009). Significant interaction effects were detected across conditions (F_1,37_ = 7.331, p = 0.01) (**Fig 3**). Post hoc paired *t*-tests showed that the reaction time for nonprototypical-complex (*mean* ± *std* = 1527ms ± 334ms) was significantly slower than nonprototypical-simple (1432 ms ± 279 ms), prototypical-complex (1422 ms ± 293 ms), prototypical-simple stimuli (1421 ms ± 300 ms).

### fMRI results

**Fig 4:**
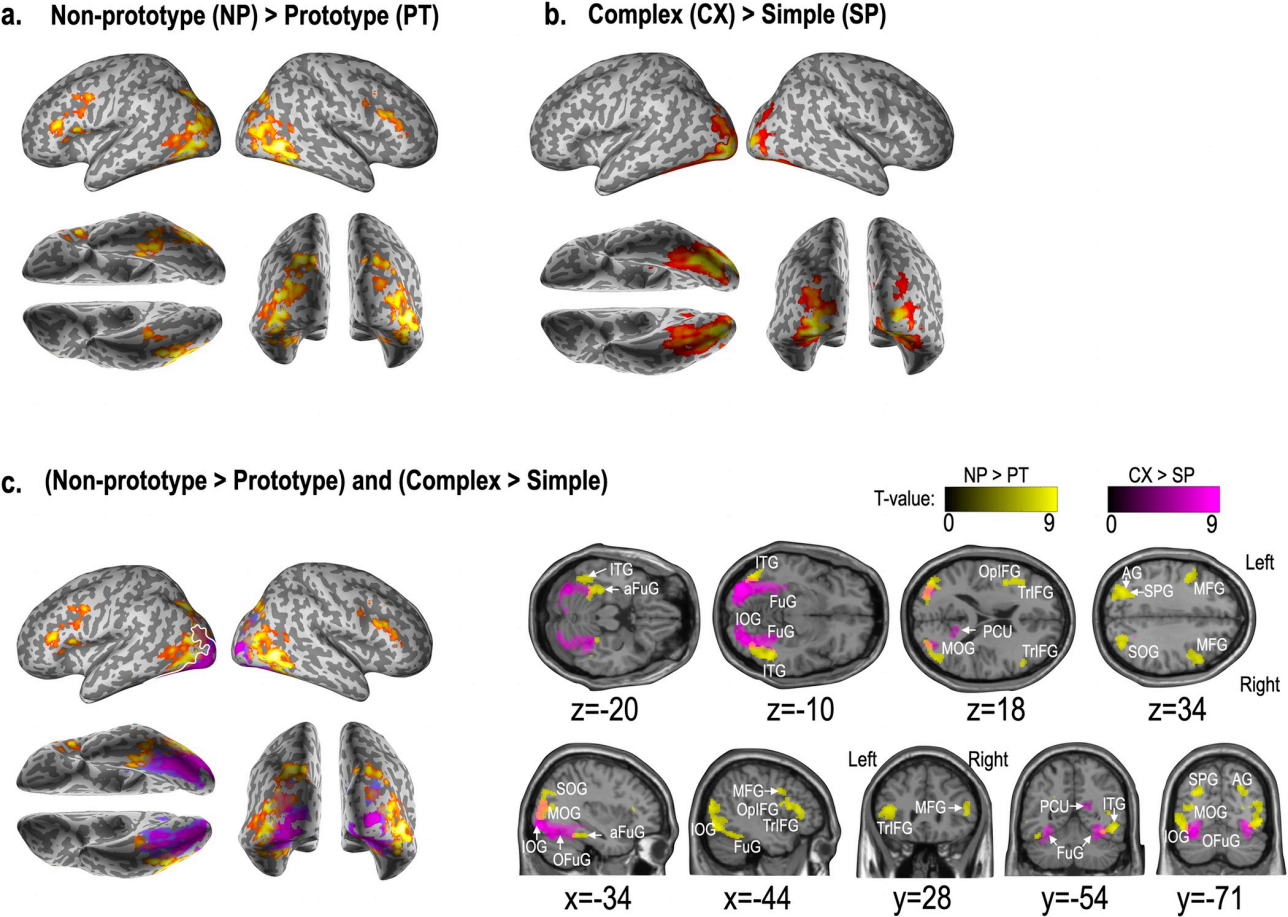
Activation maps for factor contrasts; a. non-prototypicality vs. prototypicality, b. complexity vs. simplicity, c. contrast for two conditions (i.e., prototypicality and simplicity) together. The clusters with a threshold p < 0.005 and cluster size k > 233 are displayed. SOG: Superior occipital gyrus, MOG: Middle occipital gyrus, IOG: Inferior occipital gyrus, OFuG: Cccipital part of the fusiform gyrus, aFUG: Anterior part of the fusiform gyrus, TrIFG: Triangular part of the inferior frontal gyrus, OpIFG: Opercular part of the inferior frontal gyrus, MFG: Middle frontal gyrus, PCu: Precuneus, SPG: Superior parietal gyrus, AG: Angular gyrus.

**Table 1** summarizes the fMRI results for the main and interaction effects between conditions. Compared with prototypical stimuli, non-prototypical stimuli induced greater neural activations in the bilateral superior occipital gyrus, inferior temporal gyrus, angular or superior parietal lobule, and inferior frontal gyrus. There were no significant differences in the vice versa contrast (**Fig 4A**).

**Table 1:** Brain activation corresponding to the main effects of prototypicality and all simplicity and their interaction effects.

Region	Coordinate x,y,z	Zmax	Cluster size	Region	Coordinate x,y,z	Zmax	Cluster size
*Non-prototype > Prototype*				*Complex > simple*			
L inferior occipital gyrus	-44, -78, -4	5.14	2067	R occipital pole	24, -92, -6	7.29	3083
L angular gyrus/superior parietal gyrus	-28, -74,34	4.81	-	R occipital fusiform gyrus /cerebellum exterior	30, -82, -10	6.28	-
L middle/superior occipital gyrus	-38, -76,12	4.69	-	R posterior part of the fusiform gyrus	32, -60, -14	5.33	-
L anterior part of the fusiform gyrus	-32, -32, -20	3.93	-	R inferior occipital gyrus	26, -94, -4	6.71	-
L parahippocampal gyrus	-30, -30, -18	3.40	-	R middle occipital gyrus	34, -80, 14	3.92	-
R inferior occipital gyrus	50, -68, -4	4.84	1431	L inferior occipital gyrus	-32, -86, -10	7.18	3146
R inferior temporal gyrus	48, -48, -14	4.78	-	L occipital fusiform gyrus	-22, -90, -8	6.84	-
R anterior part of the fusiform gyrus	28, -36, -18	3.66	-	L occipital fusiform gyrus/ cerebellum exterior	-32, -68, -16	6.32	-
R superior/middle occipital gyrus	28, -74,38	4.33	261	L inferior occipital gyrus	-32, -88, -10	6.87	-
R superior parietal/angular gyrus	28, -72, 40	3.49	-	L middle occipital gyrus	-30, -86, 14	4.79	-
L opercular part of the inferior frontal gyrus	-44,18,14	4.16	665	L posterior part of the fusiform gyrus	-32, -48, -18	4.23	-
L triangular part of the inferior frontal gyrus	-42,28,14	4.02	-	R precuneus	26, -60,28	3.64	260
L middle frontal gyrus	-52, 12, 36	3.31	-	*(Complex > simple)* x *(Non-prototype > Prototype)*			
R middle frontal gyrus/opercular part of the inferior frontal gyrus	48,22,24	4	475	R fusiform gyrus/Cerebellum Exterior	28, -48, -18	4.4	1301
R middle frontal gyrus/precentral gyrus	48,10,32	3.76	-	R superior/middle occipital gyrus	22, -88,18	4.01	-
				R occipital fusiform gyrus/ cerebellum exterior	32, -78, -18	3.78	-

*p* < 0.005, cluster size > 233, x, y, z: Montreal Neurological Institute coordinate, Zmax: Z maximum within a cluster, L: L, R: R. “-” in the cluster size indicates that this coordinate is a peak location that belongs to the cluster listed immediately above.

As for the simplicity/complexity, complex stimuli induced a greater level of neural activation in the bilateral inferior and middle occipital gyrus, occipital pole, occipital fusiform gyrus, and the posterior part of the fusiform gyrus. Increased involvement in the right precuneus was also observed for complex stimuli, while no increased activations were found for simple stimuli (**Fig 4B**). A noticeable spatial gradient (from the primary visual cortices, the secondary extrastriate visual cortices, and to higher-order brain regions) was found between the two main effects (**Fig 4C**).

**Fig 5:**
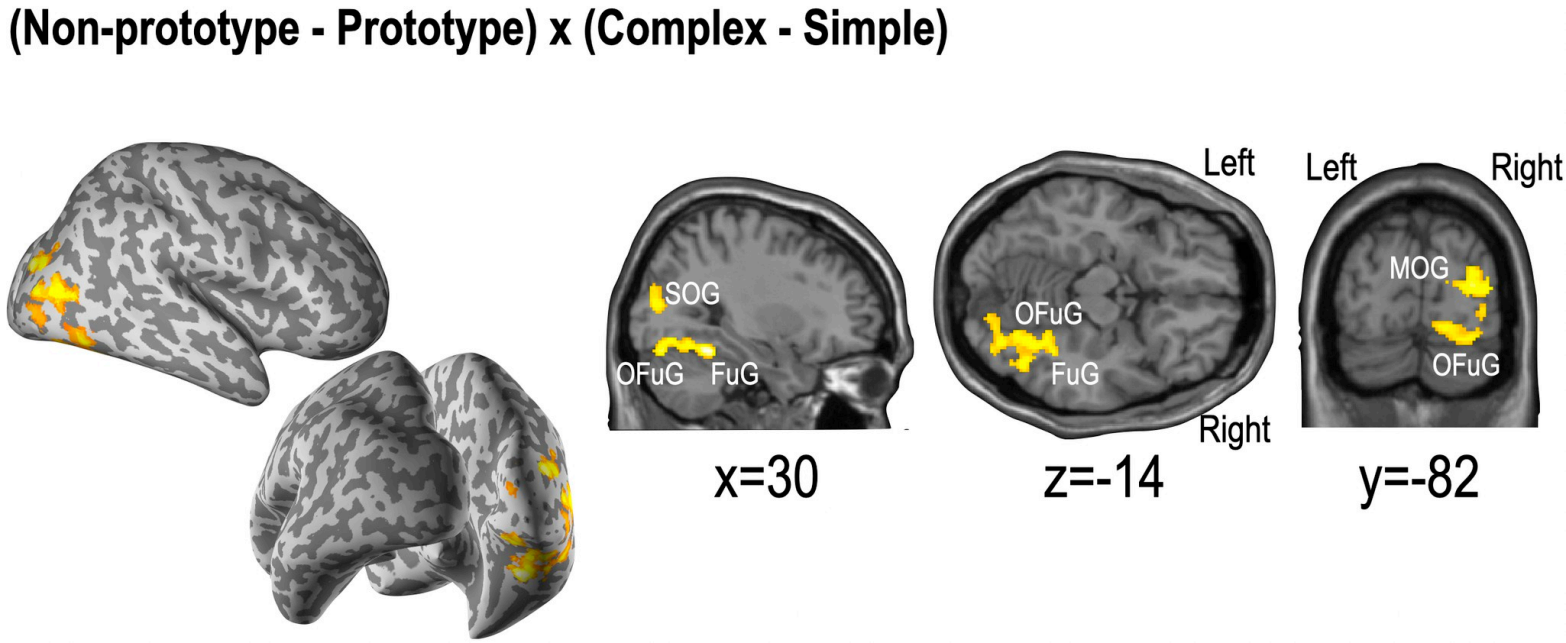
Statistical maps for interaction analysis between categories. SOG: Superior occipital gyrus, MOG: Middle occipital gyrus, OFuG: Occipital part of the fusiform gyrus, FUG: Fusiform gyrus.

The interaction effects among non-prototypical versus prototypical conditions and complex versus simple conditions were observed in the right hemisphere; the superior and middle occipital gyrus, the right occipital fusiform gyrus, and the fusiform gyrus (**Fig 5**).

## Discussion

The increasing focus on the relationship between brain processes and design is prominently reflected in recent research. Hay et al. [65] offer a perspective on using fMRI in design, highlighting its potential to unravel the cognitive underpinnings of the design process. Additionally, Rui and Gu [66] have provided a comprehensive review of EEG and fMRI studies in neuroaesthetic processing, particularly in human-computer interaction, illustrating the impact of visual brain studies on UI and UX design. Yet, the availability of neuroimaging studies in this field remains limited. Schoen et al. [67] investigated the neural correlates of product feature processing using MEG and EEG. Other fMRI studies have examined aspects of design thinking as compared to problem-solving [68], as well as product design ideation and design fixation during conceptual design phases [69–71], focusing on brain activation from the designer’s perspective. From the consumer’s viewpoint, research has delved into the neural basis of choice, particularly in making moral judgments about sustainable product options [72]. Sylcott et al. [73] explored the neural underpinnings of preference judgments concerning aesthetic and functional preferences, highlighting the role of emotional factors.

Our current study stands out in this context as it specifically investigates how the brain processes the elements of prototypicality and simplicity in product design, within the framework of design preferences. These factors are crucial in shaping consumer preferences, yet they have not been explicitly addressed in the aforementioned studies. This research contributes uniquely to the understanding of design neurocognition, shedding light on the neural mechanisms that underlie the preferences for specific visual qualities in product design.

We posited that although the effect of both prototypicality and simplicity on design preference is operated by the mechanism of fluency, the concept of fluency differs, and the brain regions involved in fluency for prototypicality should differ from those for simplicity, and vice versa.

The results from the behavioral experiment indicate the following. When examining the subjective preference rating, we found that the preference rating for prototypical (simple) stimuli tends to be higher than that of non-prototypical (complex) stimuli, but these differences were not statistically significant. However, the analysis of reaction times (RT) is significantly shorter for prototypical stimuli than non-prototypical stimuli, consistent with the findings from studies using patterns with varying prototypicality [74]. The faster reaction time is an indication of fluent processing. Based on the argument that fluency enhances liking, the data from RT would suggest that prototypical design (simple design) is preferred to non-prototypical design (complex design). As for the interaction effect, we find that the reaction time for nonprototypical-complex stimuli is significantly longer than that for all the other types of stimuli. In other words, subjects exhibit the lowest level of fluency for nonprototypical-complex designs compared to other interaction pairings.

The fMRI results show two distinct functional gradients for the preference processing of complexity (vs. simplicity) and non-prototypicality (vs. prototypicality). Specifically, we find that processing complexity recruits the occipital cortex primarily while processing prototypicality requires the involvement of the brain regions beyond the occipital cortex.

More specifically, the perception of complex designs compared to that of simple designs recruits the extrastriate visual areas as well as the primary visual cortex (i.e., occipital pole/ V1) extending lateral occipital complex. The visual information from the low level goes through the visual process sequence in the extrastriate cortex (i.e., occipital cortex/ V3, V4, V5) until overall object representations are formed [61]. In processing the complex design, it recruits visual information centers from the low level (e.g., occipital pole and lateral occipital complex) to the higher level, such as the precuneus and the occipital fusiform gyrus. Previous literature indicates that the precuneus is involved in visual processing and visuospatial imagery. This area is known to subserve shape analysis (e.g., segmentation, grouping, and surface extraction) and is associated with sensory processing rather than recognition [60]. The posterior fusiform gyrus, where V4 is located [75], is involved in the feature processing of objects [76,77]. Considering the complex design stimulated these activations, we could suggest that the complexity/simplicity factor might be the bottom-up processing in visual perception.

In contrast to the activity for the complex stimuli, non-prototypical stimuli provoke wide activations in the inferior frontal gyrus, superior or angular parietal lobule, inferior temporal gyrus, anterior part of the fusiform gyrus, parahippocampal gyrus, and secondary visual areas as compared with prototypical stimuli. The brain regions as an extension of the temporo-occipital cortices, such as the anterior part of the fusiform gyrus, parahippocampal gyrus, and inferior temporal gyrus, correspond to the higher stage of the ventral stream of visual information, called “what” process [78]. These regions are known to be involved in retrieving visual semantic information [77]. The temporo-occipital cortices have also been argued to be associated with retrieving shape information or visual semantic processing [79]. The parahippocampus is the area that involves memory encoding and retrieval [60,80,81]; visual information and memories—both semantic memories (e.g., facts and concepts) and episodic memories (e.g., autobiographical experiences related with an event)—are combined [82]. The activation in the bilateral superior parietal lobule, known as the angular gyrus, is involved in supporting semantic retrieval and attention processes. In a meta-analysis, the angular gyrus was concluded to play a role in complex information integration and knowledge retrieval [83]. The frontal regions are known to be involved in top-down semantic processing of visual information in combination with the parietal lobe mentioned above [84]–processing object features that cluster together, and retrieving stored information [85–87], and handling semantic representations within a semantic working memory system, which includes retrieving, maintaining, monitoring, and manipulating these representations [57,59,86,88]. Zanto and colleagues [58] reported that the activation of the left inferior frontal gyrus and the bilateral superior parietal lobule (i.e., a network of frontoparietal cortical regions) is a prime candidate underlying the top-down modulation. These frontoparietal circuits work in association with higher-level pathways from the visual cortices in processing non-prototype stimuli, such as the parahippocampal region, the anterior part of the fusiform, and the interior temporal gyrus.

While the complex stimuli induce increased activity at the posterior fusiform gyrus compared to the simple stimuli, non-prototypical stimuli activated the anterior fusiform gyrus more than the prototypical ones. In a study with semantic dementia, the hypometabolism in the bilateral anterior fusiform gyrus, more severe for the semantic task, suggests that the anterior fusiform gyrus is related to semantic memory [89]. Studies indicate that visual semantic memory or semantic representation is processed in the anterior fusiform gyrus [90–93]. Considering the information pathway from the anterior to posterior studied in the electrophysiology of the macaque [94,95], the brain activation regions for complexity and non-prototypicality in the preference choice suggest the hierarchical levels of the complexity and non-prototypicality. During the preference evaluation of a product, evaluation of the product concerning prototypicality is more likely to occur after the evaluation of the simplicity of the product. Although we separated these two properties in the current study, we speculate that the two properties affect the preference choice via interactions between bottom-up and top-down processing.

The interaction between prototypicality and simplicity conditions during the preference choice was mainly found in the extrastriate cortical areas in the right hemisphere, such as the right superior occipital gyrus, middle occipital gyrus, occipital fusiform gyrus, and fusiform gyrus. In those regions, the activity patterns between non-prototypicality versus prototypicality evaluations differ according to the product’s complexity (or simplicity). We also found right hemisphere dominance. Considerable neuropsychological studies in visual processing illustrated the hemispheric difference in visual processing [96,97]. The right hemisphere is dominant for representing global visual stimuli and the processing of coordination in spatial relationships [98–100]. That is, the right hemisphere is “more visually intelligent” than the left, playing the role of “interpreter” [101]. Given that during fMRI scanning, the participants were asked their preference for each stimulus, this interaction effect could be thought that global visual processing outweighed local processing for preference judgment. Global processing could be thought to be as top-down processing [102,103], which is considered to be related to the prototypicality in this study. Thus, we might be able to say that the preference judgment is biased toward the prototypicality factor over the simplicity ones. This sounds natural since, in visual perception, global processing affects the local features and visual elements [104,105]. Another explanation of the right hemisphere dominance can be the fine-coarse coding hypothesis (for language processing) [106,107]. This hypothesis posits that the right hemisphere is adept at activating a broad semantic field, particularly effective for linking concepts that are distantly related but overlap in meaning. Research has indicated that the right hemisphere plays a key role in processing texts with weak semantic constraints, whereas the left hemisphere is more involved in understanding texts with strong semantic constraints [108,109]. It has also been observed that the processing of consistent information predominantly occurs in the left hemisphere, while the processing of inconsistent information takes place in the right hemisphere [110].

There are several limitations of the current study. Although we analyzed the data from 22 participants, more data would lead to increased statistical power, thus rendering more concrete conclusions. In this study, we focused on the perception of prototypicality and simplicity under the preference judgment. Contrasting brain responses for the complexity or simplicity judgments under the context of preference judgment with those without preference judgment would reveal interactions between processing for the preference and prototypicality or simplicity. Another methodological aspect of the current study is that the product categories employed in the current study are chairs and speakers, which differ in functionality. We did not separately analyze brain responses for each type due to a small number of trials. However, the preferred choice for different types of functionality could be further researched.

This study set out to explore how the brain processes two key visual aspects of product design—prototypicality and simplicity—in the context of design preferences. Our findings reveal that although prototypicality and simplicity both influence design preferences through the perceptual mechanism of fluency, they engage distinct neural pathways. Specifically, simplicity is processed through lower-level visual pathways, while prototypicality involves higher-level semantic processing. This finding highlights that the cognitive processes for prototypicality and simplicity, governing fluency in design preferences, differ not only in concept but also in their neurobiological underpinnings.

This research offers valuable insights into the interplay between cognitive processing and aesthetic judgment in design, contributing significantly to design, marketing, and cognitive neuroscience fields. It underscores the intricate relationship between various cognitive processes and the formation of aesthetic preferences. Future research in this area could expand upon these findings, exploring how these insights into neurocognitive processing can inform modern design practices and consumer preferences, further bridging the gap between neuroscience and practical design application.
